# Abundance and Population Structure of Small Rodents in Fruit and Berry Farms

**DOI:** 10.3390/life13020375

**Published:** 2023-01-29

**Authors:** Linas Balčiauskas, Vitalijus Stirkė, Laima Balčiauskienė

**Affiliations:** Nature Research Centre, Akademijos 2, LT-08412 Vilnius, Lithuania

**Keywords:** rodents, fruit farms, population structure, relative abundance, litter size

## Abstract

Fruit and berry farms are anthropogenic habitats still inhabited by small mammals, though their presence is constantly affected by agricultural activities. Based on trapping data from 2018–2022, we analyzed the abundance and population structure of the dominant rodent species to assess changes in gender and age ratios by year and habitat, the annual and seasonal dynamics of relative abundance, and the relationship between breeding parameters and abundance. The relative abundance of the dominant species, common vole, yellow-necked mouse, striped field mouse, and bank vole, and their proportion in the investigated community varied according to year, season, and habitat. No outbreaks were recorded during the study period. The abundance of the striped field mouse exhibited a downward trend independently of habitat, while the abundance and proportions of the other three species were habitat-dependent. There was no consistent pattern between litter size and relative abundance in the same or following years. Given the ongoing conflict between biodiversity conservation in Europe and agriculture, the results contribute to a better understanding of the functioning and viability of rodent populations in fruit farms and may be used in agroecology and sustainable farming.

## 1. Introduction

The global decline in biodiversity with an increasing rate is widely recognized [1,2]. Of the many factors contributing to biodiversity loss, increasing anthropogenic pressures are considered to be the most important [3], and there is still a need to identify management measures for the conflicts between human activities and wildlife [4,5]. One of the most important questions related to small mammals is how to avoid conflicts between humans and these animals in agricultural [6] and residential areas [7].

Despite various eradication measures, small mammals remain an integral part of agricultural ecosystems [8]. Rodent damage to crops and forest plantations [9,10,11] is at its highest following outbreaks [12], which have been observed simultaneously across Europe [12]. Outbreaks are mainly linked to food supply [13], altering survival, and reproduction. The population dynamics of cyclic rodents are partly dependent on changes in reproductive parameters, which are related to climate variables [14]. However, the results on the abundance dynamics of the non-cyclic common vole show a strong effect of mortality and disease, as population growth rates were not related to climatic variables and female reproductive parameters. Therefore, the mechanism of the rodent outbreaks is not fully clear [15].

Rodent management issues are very important because of the damage they cause [14,16] and the sustainability of agriculture, which require coordinated eradication. Therefore, integrated pest management aims to minimize the use of rodenticides by recommending environmentally safer methods [17]. Data on rodent species composition and biology are essential for ecologically sound rodent management [18]. Understanding the dynamics of rodent numbers [17], their spatial structure [19,20], and reproductive patterns [21] helps to minimize the damage to agriculture by selecting the optimal timing, location, and scale of rodent management measures.

According to the FAO, there are more than 2.2 million orchards in the world, covering 53 million hectares, and giving an average orchard size of about 24 hectares [22]. In the EU, Poland had the largest area of apple orchards in 2017, expanding by 17,700 ha (23,900 ha for the EU as a whole) in 2012–2017. Traditionally, orchards are not only a source of food, but also a source of well-being for people and wildlife [23]. As a habitat with different conditions for animals than crop fields, orchards can support a wide diversity of small mammals [20,24,25]. The edge effect, which leads to a reduction in rodents in agricultural habitats in the middle of larger areas [19], has not been investigated in orchards. Our previous work demonstrating the positive role of orchards in maintaining small mammal diversity as a function of farm age and agricultural intensity [26,27] did not consider the age and sex composition of the species, which adds to the information on their adaptive capacity and viability. It has been argued that population increases are driven by survival, and not reproduction [28]. However, in the middle latitudes, reproduction intensity is certainly related to population dynamics [12,14,15]; therefore, the monitoring of pest rodent species requires knowledge of seasonal variations in abundance and the population structure and their reproduction parameters [21].

The aim of the study was to analyze the dynamics of the dominant small rodent species in fruit farms by (i) assessing the age and gender proportions by year and habitat, (ii) analyzing the annual and seasonal dynamics of the relative abundance and the proportions of the most abundant species in the community in different crops, and (iii) checking whether the proportion of breeding females and the mean litter size correlate with the abundance of the species.

## 2. Materials and Methods

### 2.1. Study Sites

**Figure 1:**
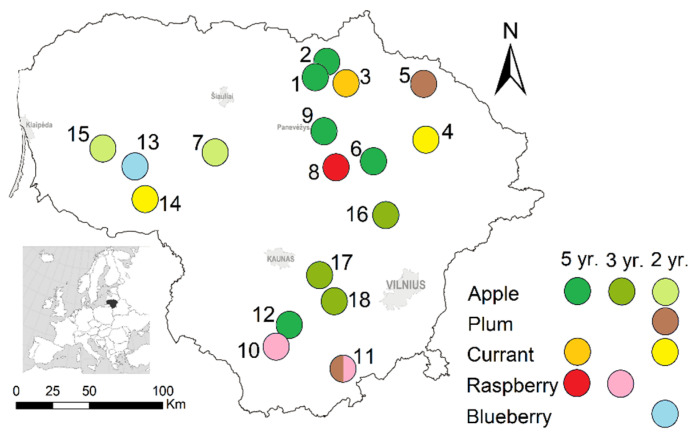
Survey sites in Lithuania, 2018–2022, represented by numbers, with indicated habitats and duration of the survey.

Eighteen areas with fruit and berry farms (63.7 ha on average) in Lithuania were surveyed between 2018 and 2022 (Figure 1). A few sites were only surveyed in 2018–2019 and were subsequently abandoned for logistical reasons (sites in the west of the country) or poor results, such as a blueberry plantation or young apple orchards, where small mammals were absent due to very intensive agricultural practices. In each area, crops were combined with control habitats, either hay meadows/non-hay meadows or forests. Additional details about the study sites are presented in [27].

### 2.2. Small Mammal Trapping: Trapping Effort and Sample Size

In 2018–2022, we snap-trapped small mammals using a standard method: trap lines of 25 traps at 5 m intervals, exposed for three days, baited with brown bread and raw sunflower oil, and checked once a day in the morning [29]. The same 7 × 14 cm standard kill traps were used in all years. The sampling unit was a three-day trapping session in a single habitat, in a given year, and at a given season. Two trapping sessions were used per year, the first in summer (June) and the second in autumn (September–October), with the exception of 2022, when small mammals were trapped only in autumn. The total trapping effort was 36,978 trapping days, 23,843 in fruit and berry farms, and 12,835 in control habitats (Table 1). 

Trapped small mammals were identified by their external features, with grey voles of the genus *Microtus* by their teeth at dissection and after cleaning skulls [30].

The age and sex of the animal were determined at dissection. Juveniles (juv), sub-adults (sub), and adults (ad) were determined by thymic atrophy, which decreases with age [31,32], and the condition of the genitalia [33,34]. The juvenile category included individuals with a fully developed thymus, but still developing reproductive organs, such as a closed vagina, a thread-like uterus, and testicles retracted into the abdomen [35]. Individuals with no signs of breeding, inactive albeit developed genitalia, and partially involuted thymuses were recognized as subadults. Breeding individuals with atrophied thymuses were classified as adults. These included overwintered individuals, males with scrotal testes and developed accessory glands, as well as pregnant or lactating females, including those with perforated vaginas [32]. Body mass was used as an additional trait for age determination.

### 2.3. Data Analyses

We analyzed the number of species in the small mammal communities, the relative abundance (number of individuals trapped per 100 trap/days), and the proportion of dominant ones of the common vole (*Microtus arvalis*), the yellow-necked mouse (*Apodemus flavicollis*), the striped field mouse (*Apodemus agrarius*), and the bank vole (*Clethrionomys glareolus*). The average values of all these indices for each trapping session (n = 215) were calculated and used as baseline data. The sex ratio and proportions of the age groups of these species were analyzed by year and habitat. Data were not transposed.

Proportions with Fisher’s 95% confidence intervals (CIs) were calculated online, using Quantitative Parasitology software, Qpweb version 1.0.15 (https://www2.univet.hu/qpweb/qp10/index.php, accessed on 10 November 2022) [36]. The significance of differences in the proportions was assessed using the online G-test calculator (https://elem.com/~btilly/effective-ab-testing/g-test-calculator.html accessed on 10 November 2022) [37].

The influence of the year, season, and habitat (categorical factors) on the relative abundance and proportions of species (dependent parameters) was assessed using GLM (generalized linear model) where the trapping effort was used as a continuous predictor to control for data variability. Model significance was determined using Hotelling’s T^2^, and the influence of categorical factors was estimated using eta-squared. A post-hoc analysis was performed applying Tukey HSD with unequal N. Before running the GLM, the normality of the distribution of the dependent parameters was checked using Kolmogorov–Smirnov’s D. The confidence level was set as *p* < 0.05. Calculations were performed with Statistica for Windows, version 6.0 (StatSoft, Inc., Tulsa, OK, USA) and PAST version 4.01 (Paleontological Museum, University of Oslo, Oslo, Norway).

We also tested whether the non-equal trapping efforts in between years and habitats affected the results. A positive correlation between the number of trap days and the number of trapped individuals and the number of registered species was found. To eliminate the influence of the unequal trapping effort, we constructed species accumulation curves using individual-based rarefaction and assessed the existence of a sample size threshold, i.e., a minimum number of individuals trapped [38,39,40]. The analysis was performed with the PAST software.

## 3. Results

### 3.1. Species Composition and Dominants 

During the five years of the survey, 1936 individuals representing 13 small mammal species were trapped (Table 2). Four species accounted for 90.8% of the trapped individuals: *M. arvalis*—28.7% (95% CI = 26.7–30.8%), *A. flavicollis*—27.9% (CI = 25.9–30.0%), *A. agrarius*—22.2% (CI = 20.4–24.1%), and *C. glareolus*—12.0% (CI = 10.6–13.5%). Insectivores accounted for 3%, and all other rodent species for 6.2% of all trapped individuals; therefore, their population structure was not analyzed further.

**Figure 2:**
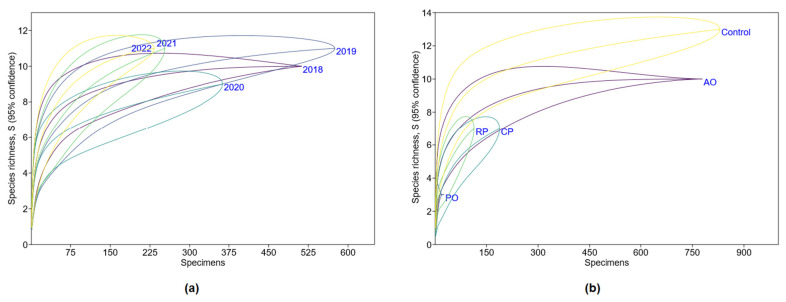
Small mammal species accumulation curves in relation to trapping effort for each year (**a**) and habitat (**b**): AO—apple orchard; PO—plum orchard; CP—currant plantation; RP—raspberry plantation.

The trapping effort varied between sites (Table 1), years, and habitats (Table 2). The number of species (r = 0.69, *p* < 0.005) and the number of individuals trapped (r = 0.72, *p* < 0.005) were positively correlated with the trapping effort. Individual-based rarefaction showed that the trapping effort was sufficient in every year (Figure 2a) and in all habitats (Figure 2b), to record the presence of no less than seven species. The number of species trapped in 2021 and 2022 was as high as in 2019, although the trapping effort decreased. Furthermore, we analyzed the abundance and population structure of the four most abundant species only.

### 3.2. Relative Abundance and Proportions of Dominant Small Rodent Species

Overall, the relative abundance and proportion of *M. arvalis*, *A. flavicollis*, *A. agrarius*, and *C. glareolus* were most strongly influenced by season (Hotelling’s T^2^ = 0.29, *p* < 0.0001) and habitat (T^2^ = 0.75, *p* < 0.0001), and less strongly influenced by year (T^2^ = 0.25, *p* < 0.05), explaining 22.3%, 8.5%, and 6.0% of variance. The trapping effort also had a cumulative effect (T^2^ = 0.25, *p* < 0.0001; eta-squared = 0.20). The univariate results varied depending on the species (Table 3).

**Figure 3:**
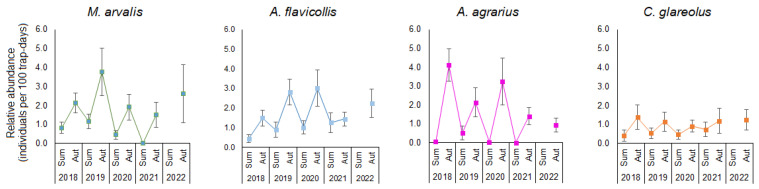
Dynamics of relative abundance of dominant rodents, 2018–2022 (data pooled).

The influence of year was significant for the relative abundance of *A. agrarius*, which showed a downward trend (Figure 3). The other two species, *A. flavicollis* and *C. glareolus*, showed an increasing trend in abundance, while *M. arvalis* showed a pattern similar with four-year cyclical changes. For these three species, the influence of the year was not pronounced, including when comparing the summer and autumn seasons separately. The differences between summer and autumn relative abundance were best expressed in *A. agrarius* (not captured in summer 2018, 2020, and 2021) and *M arvalis* (not captured in summer 2021). The other two species showed a less-pronounced increase in relative abundance in autumn, being trapped in both summer and autumn.

The average relative abundance of all four species was significantly higher in autumn than in summer. The relative abundance of *M. arvalis* increased from 0.67 to 2.44 (post hoc, *p* < 0.001), of *A. flavicollis* from 0.86 to 2.18 (*p* < 0.001), and of *C. glareolus* from 0.54 to 1.19 individuals per 100 trap days (*p* < 0.05). The same significant differences persisted from year to year (Figure 3).

**Figure 4:**
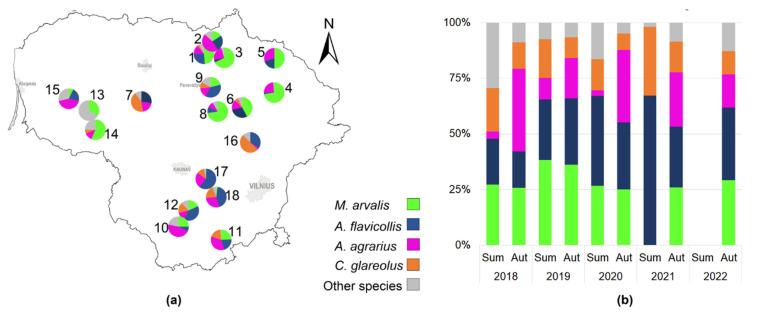
Proportions of the four dominant species in the community, irrespective of habitat: (**a**)—by site; (**b**)—by year and season.

The proportions of dominant species varied between sites (Figure 4a). Farms with old apple orchards and low agricultural activity (sites 7, 9, 12, 16, 17, and 18) had none or low proportions of *M. arvalis*, but *A. flavicollis* and *C. glareolus* were abundant in the same areas. The highest proportions of *M. arvalis* in the small mammal community were found in currant and raspberry plantations (sites 3, 4, 8, and 14). The highest percentages of *A. agrarius* were found in apple orchards and raspberry plantations under intensive farming (sites 2, 10, 11, and 15).

The effect of the year was significant for a proportion of all abundant rodent species (Figure 4b): *A. agrarius* (G = 44.3, *p* < 0.001), *A. flavicollis* (G = 44.5, *p* < 0.001), *M. arvalis* (G = 28.7, *p* < 0.001), and *C. glareolus* (G = 11.0, *p* < 0.025). The proportion of *A. agrarius* was highest in 2018 (31.1%, CI = 27.1–35.3%), and that of *M. arvalis* in 2019 (36.7%, CI = 32.8–40.9%). The highest proportion of *A. flavicollis* was recorded in 2020–2021 (35.5%, CI = 37.8–37.5% and 35.6, CI = 29.8–41.8%), compared to the lowest in 2018 (17.2%, CI = 14.0–20.7%). The highest proportion of *C. glareolus* (17.4%, CI = 12.9–22.6%) was recorded in 2021.

Seasonally, the proportions of *C. glareolus* and *A. flavicollis* decreased in autumn, the proportions of *M. arvalis* remained stable, and the proportions of *A. agrarius* increased (Figure 4b).

### 3.3. Population Structure of Dominant Small Rodent Species

At the species level (regardless of year and habitat), the male-to-female ratio was 1:1 in *A. flavicollis* and *C. glareolus*. Females were prevailing in *M. arvalis* (56.3%, CI = 52.52–60.4%; G = 17.2, *p* < 0.001), males were prevailing in *A. agrarius* (56.4%, CI = 51.6–61.0%; G = 13.3, *p* < 0.005).

**Figure 5:**
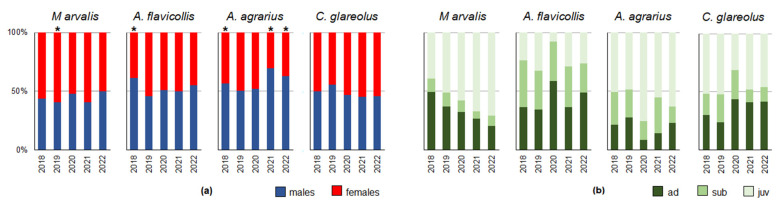
Changes in small rodent population structure by year: (**a**)—male-to-female ratio (asterisks denote significant differences based on the G-test results); (**b**)—proportions of age groups.

By year, females prevailed in *M. arvalis* between 2018 and 2021 (Figure 5a), with the highest proportion in 2021 (*p* < 0.10) and 2019 (59.5%, CI = 52.6–66.2%; G = 14.6, *p* < 0.001). *A. flavicollis* was significantly male-dominated in 2018; *A. agrarius* was significantly male-dominated in 2018, 2021 (69.4%, CI = 55.5-81.0%; G = 13.5, *p* < 0.001), and 2022; and *C. glareolus* had a sex ratio of 1:1 in all years.

At the species level (irrespective of year and habitat), juveniles prevailed in *A. agrarius* (57.2%, CI = 52.3–61.9%), *M. arvalis* (53.3%, CI = 49.0–57.5%), and *C. glareolus* (47.6%, CI = 41.0–54.3%), and adults prevailed in *A. flavicollis* (42.4%, CI = 38.2–46.7%). By year, there was a clear trend in *M. arvalis* towards a decreasing proportion of adults and an increasing proportion of juveniles (Figure 5b). Other small rodent species showed irregular changes in the proportions of age groups.

**Figure 6:**
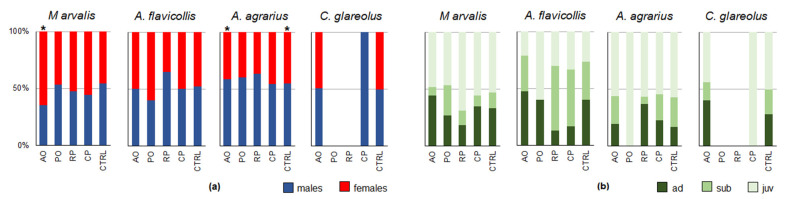
Changes in small rodent population structure by habitat: (**a**)—male-to-female ratio (asterisks denote significant differences based on the G-test results); (**b**)—proportions of age groups; AO—apple orchard; PO—plum orchard; CP—currant plantation; RP—raspberry plantation; CTRL—control habitats).

Only a few significant differences in the male-to-female ratio were confirmed between the different crops (Figure 6a). *M. arvalis* females were predominant in apple orchards (65.8%, CI = 58.1–71.1%; G = 38.0, *p* < 0.001), while *A. agrarius* males prevailed in apple orchards (58.4%, CI = 48.8–67.6%; G = 2.8, *p* < 0.05) and in the surrounding habitats (54.7%, CI = 48.1–62.3%; G = 3.8, *p* < 0.05). All other differences in the male-to-female ratio were not significant.

The highest proportions of adult individuals of *M. arvalis*, *A. flavicollis*, and *C. glareolus* were characteristic to apple orchards, and in *A. agrarius* to raspberry plantations (Figure 6b).

### 3.4. Relation of the Reproduction Parameters and Relative Abundance

**Figure 7:**
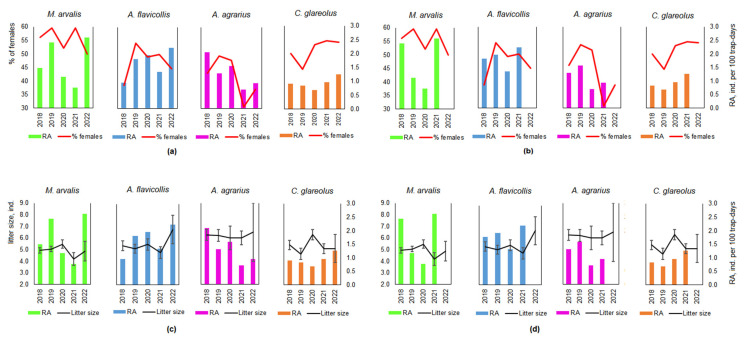
Relation of the proportion of females in the population and the average litter size to the relative abundance (RA) in the same year (**a**,**c**), and in the following year (**b**,**d**).

We found that the proportion of females in mice was more strongly correlated with the relative abundance of the species in the same year (Figure 7a), while the proportion of females in voles was more strongly correlated with the relative abundance of the species in the subsequent year (Figure 7b). In terms of correlation, the coefficients were as follows: for *M. arvalis*, r = –0.31 in the same year and r = 0.51 in the following year; for *A. flavicollis*, r = 0.45 and r = 0.16; for *A. agrarius*, r = 0.65 and r = 0.32; and for *C. glareolus*, r = 0.35 and r = 0.90, respectively.

The relationship between litter size and relative abundance in the same year (Figure 7c) was positive and most pronounced for *A. flavicollis* (r = 0.63), less so for *M. arvalis* (r = 0.30), and negative for *C. glareolus* (r = –0.50); there was no relationship for *A. agrarius* (r = –0.01). The relative abundance of the first two species declined in the following year, after litter sizes were bigger (Figure 7d): *M. arvalis* had a correlation of r = –0.85, and *A. flavicollis* r = –0.92. For *A. agrarius*, larger litters correlated with higher relative abundance in the following year (r = 0.89), whereas for *C. glareolus* the relationship was weak (r = 0.22).

## 4. Discussion

We investigated four dominant rodent species in fruit farms which belong to different trophic groups: mice *A. flavicollis* and *A. agrarius* to granivores, voles *M. arvalis* to herbivores, and *C. glareolus* to omnivores [41,42]. The relative abundance and proportion of these four species in the investigated community varied according to the year, season, and habitat with different patterns (see Table 3 and Figure 3). The downward trend in abundance of *A. agrarius* between 2018 and 2022 was independent of habitat. In contrast, the abundance and proportions of the other three species were habitat-dependent. Three species showed pronounced seasonal fluctuations in abundance, except *C. glareolus*, possibly due to its omnivory. No outbreaks were recorded during the study period, despite the fact that *M. arvalis* abundance was highest in 2019, as in other European countries [12].

Fluctuations in the abundance of *M. arvalis* can be associated with different landscape types [43], and landscape can also influence high-amplitude cycles [44]. In the same latitude as Lithuania, only half a century ago, cyclical changes in the abundance of *C. glareolus*, but not *Apodemus* mice, were confirmed with the proportion of females equal to that of males only after particularly favorable seasons [45]. Cyclic changes of vole abundances have been registered in Poland, and neighboring Lithuania, after 1986 [46]. According to data on rodent damage to forests in the 1970s, Lithuania has seen outbreaks of voles, possibly due to colder and snowier winters, and rodents were eradicated in forest nurseries [47,48].

In northern Eurasia, cyclical fluctuations of up to 500-fold over 3–5 years in voles led to changes in the proportion of pregnant females and the length of the reproductive period. These fluctuations were spatially synchronized over a distance of up to 500 km [28]. In addition to the earlier suggestion that population dynamics are driven by small mammal survival rather than reproductive rates [28], the influence of climatic variables such as temperature, precipitation, and snow cover has recently received more attention [49,50,51,52,53]. However, the climate effects coupled with environmental factors on the rodent fluctuations and their synchrony are not just limited to northern latitudes as shown in [50,53,54,55]. Winter and early spring weather parameters have been important determinants of *M. arvalis* outbreaks in east Germany [56]. Temperature and rainfall strongly influenced the reproductive patterns of the non-cyclic *M. arvalis* population in France, but not the population growth rate [15]. Recently, Spain has also reported an increase in range and outbreaks [57].

There was no consistent pattern in our data between litter size and relative abundance in the same or following years (see Figure 7c,d), and we suppose our study period is too short to analyze climatic variables. Since the beginning of the 20th century, the average annual air temperature has increased by 0.8 °C [58]. It is most pronounced during the winter and spring seasons, together with higher precipitation during the cold season and lower precipitation in April–October [59]. In 2018–2020, the average air temperature was 0.78–1.8 °C above the long-term average [60], while precipitation was below the average. However, in 2021–2022, precipitation in May to August several times exceeded average [61]. As three of the dominant species, *C. glareolus*, *M. arvalis*, and *A. flavicollis*, have been found to be capable of winter breeding in their natural habitats in Lithuania [62,63,64], winter conditions may also influence population dynamics on fruit tree farms. 

Winter conditions directly limit small mammal survival through changes in subnivean space [65] and access to food resources [66,67,68]. In agricultural areas, food availability for small mammals is regulated not only by climate, but also by a variety of other factors, such as the presence of hedgerows and seed-rich strips [69], the size and fragmentation of fields [70], farming practices [26,71], the use of crop-protection products [72], and the farm’s proximity to the natural habitat [19]. Some of these factors also affect the social characteristics of small mammal populations, such as gender and age structure or reproductive status [73,74].

Despite recent studies on small rodents in agroecosystems [18,75,76,77], fruit farms are one of the least addressed topics [24,25,78]. Our previous publications [26,27,79] have not addressed aspects of small rodent population structure, annual and seasonal dynamics of their abundance, and the correlation of relative abundance with reproductive parameters. These results therefore contribute to a better understanding of the functioning and viability of rodent populations in less-studied habitats under anthropogenic pressure, such as fruit farms. Given the ongoing conflict between biodiversity conservation in Europe and agriculture [80], the results can be used in agroecology [81] and sustainable farming.

## Figures and Tables

**Table 1:** Site-based trapping efforts and main results, 2018–2022 (AO—apple orchard; PO—plum orchard; CP—currant plantation; RP—raspberry plantation; BP—blueberry plantation; MM—mowed meadow; NM—non-moved meadow; F—forest; AG—agricultural areas; TS—number of trapping sessions; TD—number of trap days; S—species richness (number of species); N—number of individuals caught).

Site	Crop and Control Habitat	TS	TD	S	N	Site	Crop and Control Habitat	TS	TD	S	N
1	AO, MM, NM	18	4050	9	113	10	RP, MM	10	1050	6	28
2	AO, MM	18	4050	10	223	11	RP, PO, F, AG	12	900	5	87
3	CP, MM	18	4050	6	106	12	AO, MM	28	4908	8	177
4	CP, MM	8	900	5	109	13	BP, MM	6	675	2	3
5	PO, NM	8	600	3	20	14	CP, MM	8	795	6	16
6	AO, MM	18	4050	9	268	15	AO, MM	8	900	6	14
7	AO, NM	8	900	8	173	16	AO, MM	10	750	6	73
8	RP, MM, NM	18	1350	6	106	17	AO, F	10	2250	6	133
9	AO, MM, NM	18	4050	8	191	18	NM. AG	10	750	7	95

**Table 2:** Breakdown of small mammal species composition in fruit and berry farms, 2018–2022, for each year and habitat (AO—apple orchard; PO—plum orchard; CP—currant plantation; RP—raspberry plantation; CTRL—control habitats).

Species	2018	2019	2020	2021	2022	AO	PO	CP	RP	CTRL
Common shrew (*Sorex araneus*)	17	8	2	5	10	6	0	5	2	29
Pygmy shrew (*S. minutus*)	2	9	1	1	2	3	0	1	0	11
Water shrew (*Neomys fodiens*)	0	0	0	1	0	0	0	0	0	1
Northern birch mouse (*Sicista betulina*)	0	0	0	0	2	0	0	0	0	2
House mouse (*Mus musculus*)	3	2	0	3	3	3	0	2	2	4
Striped field mouse (*Apodemus agrarius*)	159	93	94	49	35	117	5	44	30	234
Yellow-necked mouse (*A. flavicollis*)	88	168	118	90	76	276	5	6	23	230
Harvest mouse (*Micromys minutus*)	4	2	6	1	5	5	0	0	1	12
Bank vole (*Clethrionomys glareolus*)	68	64	32	44	24	108	0	1	0	123
Water vole (*Arvicola amphibius*)	0	1	0	0	0	0	0	0	0	1
Common vole (*Microtus arvalis*)	133	211	92	52	68	220	16	129	55	136
Root vole (*M. oeconomus*)	20	5	17	3	1	19	0	0	1	26
Short-tailed vole (*M. agrestis*)	18	12	1	4	7	21	0	0	0	21
Total number of individuals, N	512	575	363	253	233	778	26	188	114	830
Number of species, S	10	11	9	11	11	10	3	7	7	13
Trapping effort, trap days	8880	8748	7875	7650	3825	17,568	600	3850	1675	12,835

**Table 3:** Influence of year, season, and habitat on the relative abundance (RA) and proportions (P%) of the dominant small rodent species: F and *p* breakdown of the univariate GLM results; NS – not significant.

Species	Index	Year	Season	Habitat
*M. arvalis*	RA	F = 0.77, NS	F = 9.63, *p* < 0.005	F = 2.26, *p* < 0.05
	P%	F = 0.89, NS	F = 0.36, NS	F = 2.33, *p* < 0.05
*A. flavicollis*	RA	F = 1.06, NS	F = 10.14, *p* < 0.002	F = 4.73, *p* < 0.001
	P%	F = 3.08, *p* < 0.02	F = 0.04, NS	F = 3.56, *p* < 0.001
*A. agrarius*	RA	F = 2.58, *p* < 0.05	F = 35.29, *p* < 0.001	F = 0.57, NS
	P%	F = 1.92, NS	F = 29.62, *p* < 0.001	F = 1.83, NS
*C. glareolus*	RA	F = 0.32, NS	F = 0.95, NS	F = 5.41, *p* < 0.001
	P%	F = 1.37, NS	F = 0.21, NS	F = 4.57, *p* < 0.001

## Data Availability

This is ongoing research; therefore, data are available from the corresponding author upon request.
